# The problematic Cambrian arthropod *Tuzoia* and the origin of mandibulates revisited

**DOI:** 10.1098/rsos.220933

**Published:** 2022-12-07

**Authors:** Alejandro Izquierdo-López, Jean-Bernard Caron

**Affiliations:** ^1^ Ecology and Evolutionary Biology, University of Toronto, Toronto, Ontario, Canada, M5S 3B2; ^2^ Earth Sciences, University of Toronto, Toronto, Ontario, Canada, M5S 3B2; ^3^ Royal Ontario Museum, Toronto, Ontario, Canada, M5S 2C6

**Keywords:** Cambrian, Burgess Shale, arthropods, mandibulates, palaeoecology, taphonomic bias

## Abstract

The origin of mandibulates, the hyperdiverse arthropod group that includes pancrustaceans and myriapods, dates back to the Cambrian. Bivalved arthropod groups such as hymenocarines have been argued to be early mandibulates, but many species are still poorly known, and their affinities remain uncertain. One of the most common and globally distributed Cambrian bivalved arthropods is *Tuzoia*. Originally described in 1912 from the Burgess Shale based on isolated carapaces, its full anatomy has remained largely unknown. Here, we describe new specimens of *Tuzoia* from the Canadian Burgess Shale (Wuliuan, Cambrian) showcasing exceptionally preserved soft tissues, allowing for the first comprehensive reconstruction of its anatomy, ecology and evolutionary affinities. The head bears antennae and differentiated cephalic appendages. The body is divided into a cephalothorax, a homonomous trunk bearing *ca* 10 pairs of legs with heptopodomerous endopods and enlarged basipods, and a tail fan with two pairs of caudal rami. These traits suggest that *Tuzoia* swam along the seafloor and used its spinose legs for predation or scavenging. *Tuzoia* is retrieved by a Bayesian phylogenetic analysis as an early mandibulate hymenocarine lineage, exemplifying the rapid diversification of this group in open marine environments during the Cambrian Explosion.

## 1. Introduction

The origin of mandibulates, the arthropod group that encompasses myriapods and pancrustaceans, dates back to the Cambrian period [1–3] but the early evolution of this group remains a contentious issue [4–6]. Bivalved arthropods, a polyphyletic group characterized by their cephalothoracic carapace subdivided into two valves, have been central to this debate. Some bivalved crustaceomorph larvae from Orsten deposits have been considered stem-mandibulates [7], but also stem-pancrustaceans or pancrustaceans [8]. The hymenocarines, another group of Cambrian bivalved arthropods, have been previously considered early euarthropods [9–11], but have recently been reinterpreted as early mandibulates based on the presence of putative mandibles and reduced post-mandibular appendages (i.e. maxillae) found in several species [4,5,12]. These interpretations are providing new views on the early evolution of this group, although many questions remain [12–14].

A challenge in the study of bivalved arthropods comes from the lack of soft tissues for several species, in which the carapace is often the only structure preserved [15,16]. The bivalved arthropod *Isoxys* has been known for over a century [17] almost entirely from isolated carapaces distributed across the globe [18], but its phylogenetic affinities and ecology remained unknown for more than 100 years until further soft tissues were recovered, concluding that *Isoxys* is a stem-group euarthropod with a pelagic lifestyle [19–21]. A similar case to *Isoxys* is the bivalved arthropod *Tuzoia* Walcott 1912*. Tuzoia* was one of the original species described from the Cambrian (Wuliuan) Burgess Shale, known only from its distinct reticulated large carapace valves and variable spines [22,23]. Since then, *Tuzoia* has been found in numerous Burgess Shale-type deposits, representing one of the most widespread arthropods of the era [24,25]. More than 20 species of *Tuzoia* have historically been recognized, many since synonymized or with open nomenclature [25,26]. These species were diagnosed based mainly on variations in the number, length and positioning of spines along the carapace and their geographical distribution, making *Tuzoia* one of the most diverse genera of Cambrian arthropods. Despite its ubiquity, the preservation of other soft tissues has been extremely scarce. Eyes and eye stalks have been identified in some specimens [26,27], but their exact position and morphology remain ambiguous [27,28]. Poorly preserved antennae [25,26] and legs [29–31] have also been reported, but lack detailed information on their morphology and position, preventing a full reconstruction of *Tuzoia*. Based on new specimens with exceptionally preserved soft tissues from the Cambrian Burgess Shale formation, we present the most comprehensive interpretation of the morphology, phylogenetic affinity and ecology of *Tuzoia* to date.

## 2. Methods

### 2.1. Fossil material and locality

This study is based on 11 individuals of *Tuzoia* preserving soft tissues, four of which have been published [26,29]. All specimens, possibly representing three separate species (electronic supplementary material, table S1), are deposited at the Royal Ontario Museum Invertebrate Palaeontology section (ROMIP). The total number of specimens of *Tuzoia* in the ROMIP collections is close to 400, but only specimens presenting soft tissues, besides the carapace, were included in this study. Three specimens come from classical Burgess Shale localities in Yoho National Park, while the remaining are from more recently explored localities in Kootenay National Park (electronic supplementary material, table S1). Specimens are preserved as aluminosilicate two-dimensional compressions [32]. Carapace valves may show deformation due to compression, which is most visible on the lateral edges of the carapace [33]. Specimens were photographed under direct and cross-polarized light under dry and wet conditions. Several specimens were prepared using an air-scribe, removing part of the matrix or the carapace to uncover soft tissues underneath. A list of the current species of *Tuzoia* is included in the electronic supplementary material, tables S2 and S3.

### 2.2. Phylogenetic and morphometric analysis

A morphospace for carapace shapes was built using the R (V4.1.2) packages *Momocs* [34] and *geomorph* [35] (electronic supplementary material, *Analysis 2.1*). The morphospace is based on the external outlines of singular carapace valves in lateral view of multiple bivalved arthropods belonging to the Isoxyidae and Hymenocarina, traced from previously published images. The analysis contained 20 genera and 63 shapes, with most genera being represented by at least two shapes (see electronic supplementary material, Files). Hymenocarines were classified into subgroups (e.g. waptiidae). Each valve outline was reconstructed through an elliptical Fourier analysis under 25 harmonics. A morphospace was created through a principal component analysis of the carapace shapes to examine variations in shape and an evaluation of the affinities of *Tuzoia* was tested through a K-means analysis (electronic supplementary material, *Analyses 2.1* and table S4).

A phylogenetic analysis was performed based on a modified version of a previously published panarthropod morphological matrix [14,36]. This dataset, referred to as dataset 1, was pruned to include only euarthropod and stem-euarthropod species and applicable characters. New hymenocarine characters and taxa were included from previous publications [37], as well as characters related to the frontal appendages from a different dataset [38], to a total of 113 taxa (electronic supplementary material, table S5) and 300 characters. All characters were discrete, binary or multi-state, and equally weighted. A Bayesian analysis was performed with the Mk model and gamma-distributed rate variation in MrBayes V3.2 [39]. The analysis employed two runs with four chains for 1.5 × 10^7^ generations, sampling every 100 generations, with 20% of the initial samples discarded as burn-in. Convergence was evaluated with Tracer V1.7.1 [40]. The size of the eyes (character 7) was measured on specimens of *Tuzoia*, and several species of isoxyids and hymenocarines (electronic supplementary material, *Analyses 2.2*). The same analysis was run using an alternative dataset with a more conservative coding regarding the anatomy of *Tuzoia*, leaving all characters of uncertain affinity as unknown and homologizing the head of *Tuzoia* to isoxyids, referred to as dataset 2 (electronic supplementary material, *Discussion 3.1*). A treespace visualization was created with the R packages *ape* [41] and *phangorn* [42], which shows the number of trees in which *Tuzoia* and mandibulates form a monophyletic group (electronic supplementary material, *Analyses 2.3)*.

## 3. Results

*Systematic Palaeontology*

Phylum Arthropoda von Siebold 1848

Order Hymenocarina Clarke, 1882 (emended Raymond 1935)

Family Tuzoiidae Raymond, 1935

Taxa included: *Tuzoia* Resser 1929, *Duplapex* Ma *et al*. 2021.

Emended diagnosis (from [28]): hymenocarine; valves symmetrical in the sagittal axis, generally amplete to postplete. Valves have at least an anterior and posterior carapace process, a postero-ventral spine and reticulate ornamentation.

Genus *Tuzoia* Walcott, 1912

Type species: *Tuzoia retifera* Walcott, 1912, Middle Cambrian Burgess Shale (Wuliuan), British Columbia, Canada.

Species included: *T. australis*, *T. bispinosa*, *T. burgessensis*, *T. canadensis*, *T. guntheri*, *T. jianheensis*, *T. lazizhaiensis*, *T. manchuriensis*, *T. multispinosa*, *T. polleni*, *T. retifera*, *T. sinensis*, *T. tylodesa*.

Emended diagnosis (from [26]): Tuzoiid with an ocular segment extending beyond the carapace margin and bearing a pair of elongated peduncular lobes. Carapace bearing a spinose lateral ridge. A mid-posterior spine (mps) is present in most species. Additional small marginal spines are present in some species, mostly on the ventro-posterior margin of the carapace. Endopod heptopodomerous with an elongated basipod: distalmost podomere is claw-shaped, and the second distalmost podomere always bears a series of spines. At least the first two pairs of legs bear spines on each podomere. The body terminates into two pairs of caudal rami, one on top of the other, each pair fused into a singular truncate paddle.

*Description*

The following description is a composite of several species of *Tuzoia* (see electronic supplementary material, table S1).

**Figure 1.**
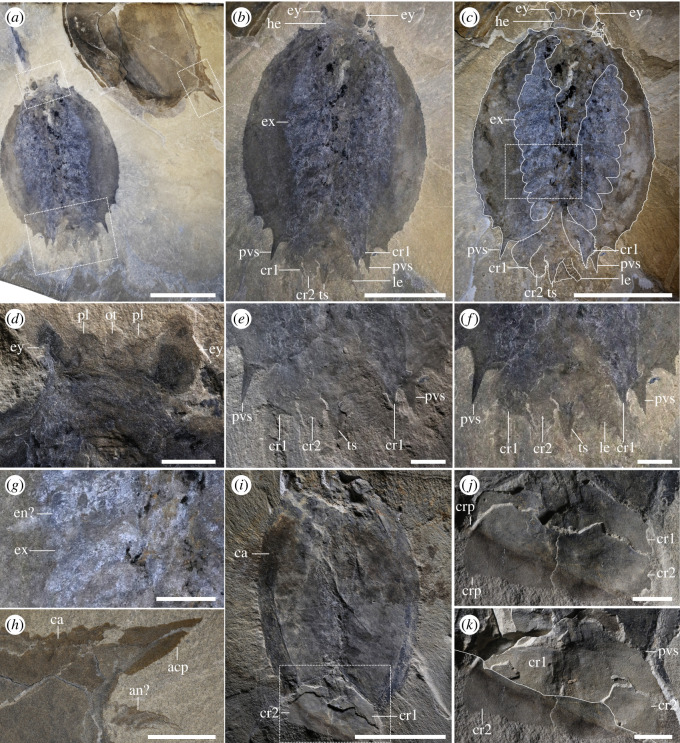
*Morphology of Tuzoia, Marble Canyon specimens*. (*a*) Two specimens (ROMIP 66176.1 and ROMIP 66176.2, *T. cf. burgessensis*); (*b*) full body in ventral view of ROMIP 66176.1 with camera-lucida interpretation (*c*), showing the head with stalked eyes and peduncular lobes (*d*), close-up of the tail fan (*e,f*) and close-up of the legs in wet condition (*g*); close-up of the potential antenna in ROMIP 66176.2; (*i*) specimen in dorsal view (ROMIP65087, *T. cf. burgessensis*), with close-ups of the tail fan: part (*j*) and a composite of part and counterpart (*k*). Scales: (*a–c,i*) = 50 mm; (*d–h,j–k*) = 10 mm. Abbreviations: acp, anterodorsal carapace process; an, antenna; ca, carapace; crp, caudal rami process; cr1-2, first or second pair of caudal rami; en, endopod; ex, exopod; ey, eye; he, head; le, leg; ot, ocular tergite; pl, peduncular lobe; pvs, postero-ventral spine; ts, terminal segment.

*General habitus*: the previous minimum recorded length was 9.8 mm from *T. bispinosa* from the Kaili biota [42], and the maximum recorded length was 170–180 mm from *Tuzoia sp*. from Bohemia [43]. Similar large specimens are known at the Burgess Shale, with *T. cf. burgessensis* from the Marble Canyon locality reaching *ca* 180 mm (ROMIP 63709). Based on this and specimens preserved with a tail fan from the same locality (figure 1*a–f,i–k*; electronic supplementary material, figures S1 and S2), and assuming that the ratio between the length of the carapace and tail fan remains constant, the biggest known *Tuzoia* could reach *ca* 210 mm in length.

**Figure 2.**
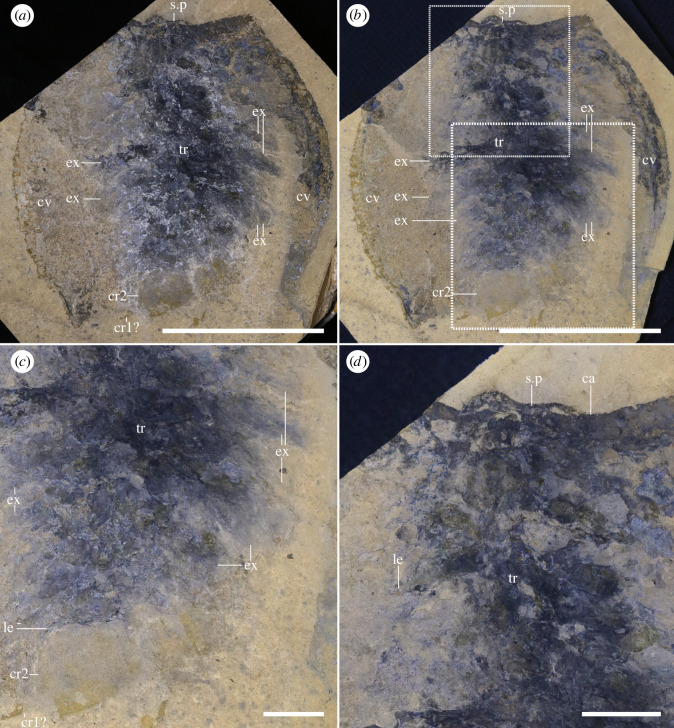
*Full body specimen with soft tissues and caudal rami*. (*a,b*) Full specimen in ventral view (ROMIP66300, *T. sp*) under dry (*a*) and wet (*b*) conditions, showing traces of potential exopods and one pair of caudal rami, highlighted in (*c*), and a close-up of the anterior section of the body, with a sclerotized plate (*d*), Scales: (*a,b*) = 50 mm; (*c,d*) = 10 mm. Abbreviations: cr1-2, caudal rami pair 1 or 2; cv, carapace valve; ex, exopod; le, leg; s.p, sclerotized plate; tr, trunk.

**Figure 3.**
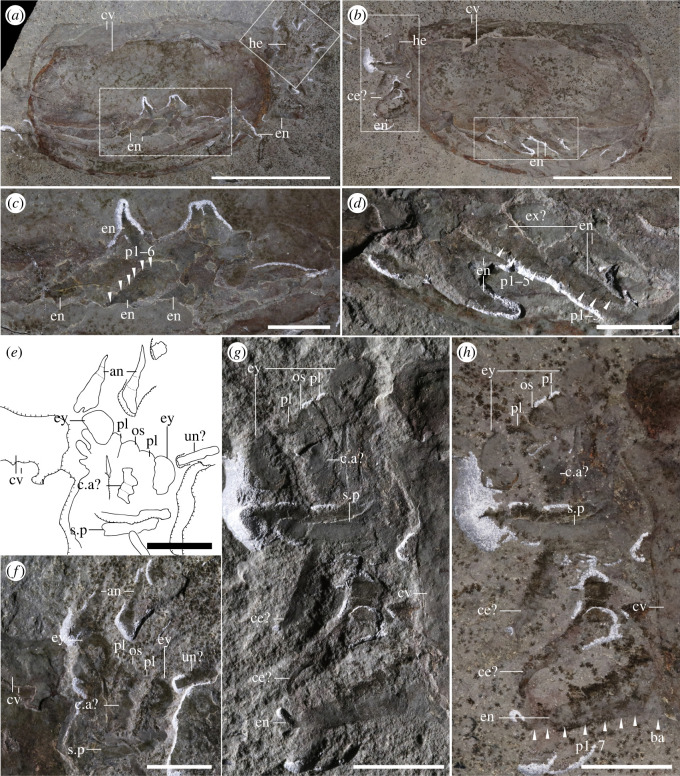
*Specimen showing legs and head*. Full specimen in lateral view (ROMIP59978, *T. retifera*): part (*a*), and counterpart (*b*), (*c*) close-up of (*a*) showing the terminal podomeres of several legs; (*d*) close-up of (*b*) showing the terminal podomeres of at least four legs; (*e,f*) close-up of (*a*) showcasing the head and antenniform appendages and camera-lucida interpretation (*e*); (*g,h*) close-up of (*b*), showing the frontal side of the specimen in dry (*g*) and wet (*h*) conditions. Scales: (*a,b*) = 50 mm, (*c–h*) = 10 mm. Abbreviations: an, antenna; c.a, cephalic appendage; ce, cephalothoracic leg; cv, carapace valve; en, endopod; ex, exopod; ey, eye; he, head; os, ocular sclerite; p*, podomere; pl, peduncular lobe; s.p, sclerotized plate.

*Head and associated structures*: the head extends beyond the anterior edge of the carapace (figures 1*a–d*; electronic supplementary material, figures S1 and S2) and bears a series of associated structures (figures 1, 2 and 3): one pair of unsegmented elongated lobe-like projections between the eyes, interpreted as peduncular lobes (figures 1*a–d*, 3*e–h*; electronic supplementary material, figure S3), and a rounded structure present between the peduncular lobes, a potential ocular sclerite (or ocular tergite, see *Discussion*) (figure 1*d* and 3*e–h*). Several specimens show an elongated, bilaterally symmetrical structure with an invaginated anterior margin present on the ventral side of the head (figures 2*a,b,d*; electronic supplementary material, figure S3e–h). The preservation of this element is similar to the carapace, indicating that it was strongly sclerotized and is therefore referred to as a sclerotized plate (see *Discussion* for potential affinities).

*Eyes*: eyes are hemiellipsoidal, as long or longer than 5% of the total body length and attach to short, thick eye stalks that extend along the lateral margins of the head (figures 1*a–d*, 3*e–h*; electronic supplementary material, figures S1 and S3). One specimen shows a differentiated outer ring (electronic supplementary material, figure S4c) which may be indicative of a separation between the lenses and crystalline cones and the remaining portion of the eye [44].

*Antennae*: Several specimens show one pair of antenniform structures approximately double the length of the eye (figures 1*h*, 3*e,f*; electronic supplementary material, figure S4c). Their segmentation and exact morphology remain ambiguous, but most probably represent a pair of first antennae.

**Figure 4.**
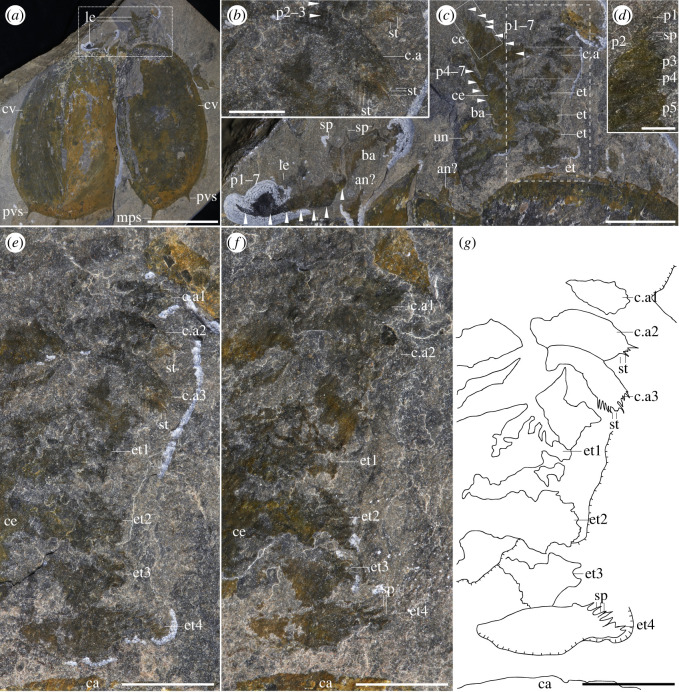
*Cephalothoracic area of Tuzoia.* (*a*) Full specimen (ROMIP57446, *T. canadensis*) showing two valves in dorsal view and soft tissues located frontally (*b,c*). These include a differentiated cephalic appendage, also shown in a close-up (*b*), a series of cephalothoracic legs, a trunk leg with an enlarged basipod bearing spines and two potential antennae; (*d*) close-up of the distalmost podomeres in a cephalothoracic leg, bearing spines; (*e*) close-up of the cephalic appendage and the endites, including part (*e*), counterpart (laterally mirrored) (*f*) and camera-lucida interpretation of the part (*g*). Note how the cephalic appendage is smaller in size and has curved setae, when compared to the endites. Scales: (*a*) = 50 mm; (*c,e–g*) = 10 mm; (*b,d*) = 2.5 mm. Abbreviations: an, antenna; ba, basipod; c.a, cephalic appendage; ca, carapace; ce, cephalothoracic leg; cv, carapace valve; et, endite; le, leg; mps, medio-posterior spine; p*, podomere; pvs, postero-ventral spine; sp, spine; st, setae; un, unknown.

*Post-antennal cephalic appendage*: one specimen shows a three-segmented structure close to the head, with terminal lobes bearing thin curved setae (figure 4*b,e–g*), morphologically distinct from the antennae or legs. These cephalic appendages may correspond to a mandibular palp or to maxillae (see *Discussion*).

*Cephalothoracic legs*: the first one to two pairs of legs are differentiated from the following trunk legs and are here interpreted as cephalothoracic legs. Their endopod is stout, subdivided into seven disc-shaped podomeres, wider than longer, which become smaller distally (figures 4*c,d*; electronic supplementary material, figure S4b,d). The distalmost podomere is shaped into a claw (figures 4*c,d*; electronic supplementary material, figure S4b,d). The second distalmost podomere is short and bears 6–7 curved to straight spines; the distalmost spines are longer than the remaining ones and are positioned almost parallel to the terminal claw (figure 4*d*). The third and fourth podomeres have a straight to slightly convex ventral margin and bear an indeterminate number of small spines, while the remaining fifth to seventh podomeres bear a large straight spine located medially, perpendicular to their ventral surfaces (figure 4*c,d*). The basipod is highly elongated and bears a series of well-developed elongated endites that terminate into spinose processes (figure 4*c–g*). At least two to four endites are visible, and taking the length of the basipod as a reference, we infer a total of 5 to 6 endites. The presence of an exopod in these legs is ambiguous.

**Figure 5.**
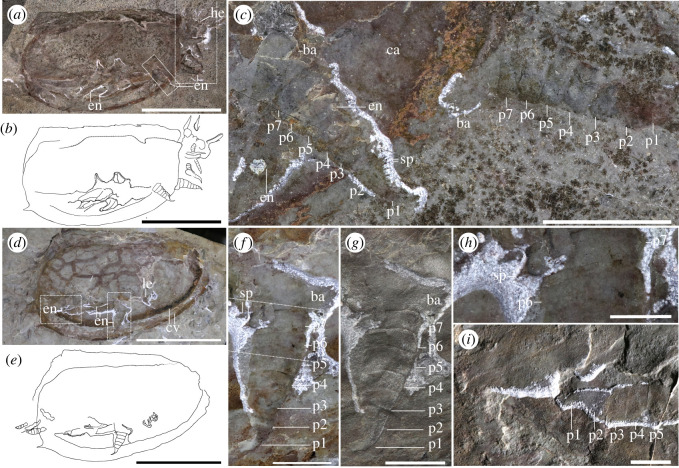
*Thoracic legs of Tuzoia*. (*a*) Full specimen in lateral view (ROMIP59978, *T. retifera*), with camera-lucida interpretation of the part and counterpart superimposed (*b*) and a close-up on two trunk legs, showing podomeres and basipod (*c*); (*d*) full specimen in lateral view (ROMIP64835, *T. retifera*) with camera-lucida interpretation (*e*), and several close-ups: a trunk leg under wet condition (*f*) and dry condition with direct light (*g*), a further close-up of (*f*), showing the spines of the proximalmost podomere and basipod (*h*) and a close-up of (*d*) showing two trunk legs ending in claw-like podomeres (*i*). Scales: (*a,b,d,e*) = 50 mm; (*c*) = 10 mm; (*f,g,i*) = 5 mm; (*h*) = 2.5 mm. Abbreviations: ba, basipod; ca, carapace; cv, carapace valve; en, endopod; he, head; p*, podomere; sp, spine.

*Trunk and legs*: the trunk of *Tuzoia* is almost entirely covered by the carapace; it is fusiform and large, and its size increases medially (figures 1*a–c*, 2; electronic supplementary material, figure S1). Segmentation is not discernible from the specimens available, but taking the size of the legs as approximations, there are most probably 10 pairs of biramous legs which become shorter towards the anterior and posterior side of the body (figures 1*a–c*, 2; electronic supplementary material, figure S1). Legs are always shorter than the carapace height, so only the anteriormost and posteriormost legs can extend beyond the edges of a closed carapace (figures 3*a–d*, 5*a–c*; electronic supplementary material, figure S4f,g). Elliptic impressions suggest the presence of paddle-shaped exopods, indicating that all legs were most probably biramous (figures 1*b,c,g* and 2) but their exact morphology remains ambiguous. All trunk legs are most probably almost identical (figures 3*c,d* and 5*a–e*; electronic supplementary material, figure s4*f–g*); their endopods have seven disc-shaped podomeres of a similar size. The distalmost podomere is shaped into a claw (figures 4*c*, 5*c,f,g,i*). The second distalmost podomere is usually longer than the rest, and bears elongated, straight to anteriorly slightly curved spines along its ventral margin (figure 5*c*). The remaining podomeres are devoid of spines (figures 4*c*, 5*c,f–i*), excluding the seventh distalmost podomere, which bears a medial straight spine on its ventral surface (figure 4*c* and 5*h*). The basipod is elongated, unsegmented and bears a singular spine proximal to the seventh podomere (figures 4*c* and 5*a–h*).

*Tail fan*: the trunk becomes thinner posteriorly, extending slightly beyond the posterior margin of the carapace (figures 1*a–c,e,f*; electronic supplementary material, figure S1) and terminates into an elongated structure. This structure could represent the two posteriormost trunk segments or a telson, and bears two pairs of paddle-like appendages originating dorsolaterally, covering the aforementioned segments (figure 1*i–k*). Both pairs of paddle-like appendages are morphologically similar, with the first extending above the second one; each pair is partly fused into a singular oval paddle that truncates posteriorly, with its outer margins extending posteriorly into small straight to curved processes, more prominent in the first pair of paddles than the second pair (figures 1*a–c,e,f,i–k* and 2*a–c*; electronic supplementary material, figures S1,2). These structures are referred to as caudal rami (but see *Discussion*).

## 4. Discussion

### 4.1. Previous and current interpretations of the morphology of *Tuzoia*

**Figure 6.**
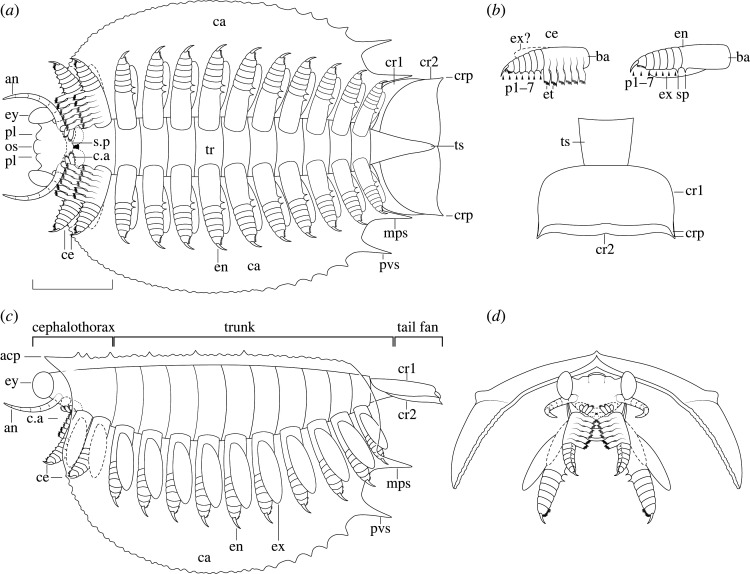
*Diagrammatic reconstruction of Tuzoia.* Diagrammatic reconstruction of *Tuzoia* in ventral view (*a*), with the carapace corresponding to *T. burgessensis,* this view is a diagrammatic reconstruction that emphasizes the boundary between the cephalothorax and the trunk. The orientation of the cephalothoracic appendages emphasizes this differentiation, but their orientation was probably closer to that portrayed in the lateral and anterior view; (*b*) details of the cephalothoracic and trunk legs in lateral view and tail fan in dorsal view; (*c*) lateral view; (*d*) frontal view. Abbreviations: as in previous figures. Courtesy of Brittany Cheung.

**Figure 7.**
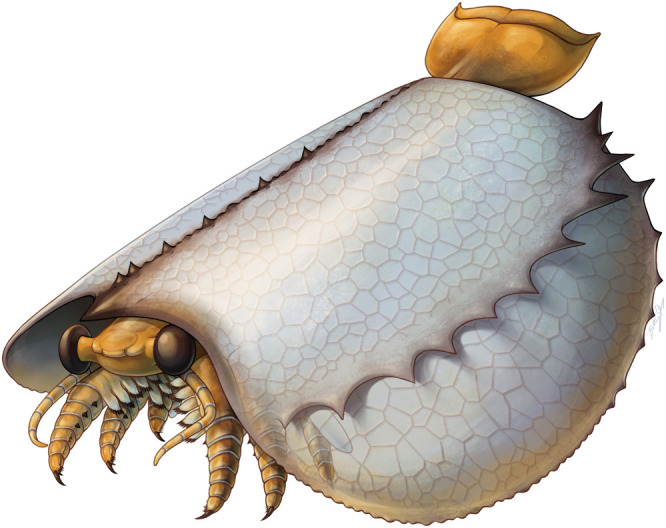
*Artistic reconstruction of Tuzoia.* Carapace based on *T. burgessensis.* Courtesy of Brittany Cheung.

The new morphological information obtained from Burgess Shale soft tissues facilitates a new reconstruction of this genus (figures 6 and 7) and confirms, complements or refutes previous interpretations about *Tuzoia* and tuzoiids as a whole. Specimens preserving eyes had been previously reported from the Balang Formation [25], the Emu Bay Shale [27], the Burgess Shale [26] and the Guanshan Biota [45]. Eye peduncles have been described as long, at least three times longer than the eye itself [26] and annulated [25,28], being one of the main diagnostic features of Tuzoiidae [28]. Several specimens (figures 1*d*, 3*e–h*; electronic supplementary material, figure S3c), as well as previously published material (Plate 6: Figs. 1 and 2 in [27]), show that the eyes are closely attached to the cephalon, a condition similar to that in *Waptia* [12] and that the eye peduncles are just slightly bigger than the eye itself. Elongated eye peduncles reported in previous descriptions are probably disassociated from the head (Fig. 23: 1–4 in [26]) and show tissues transversal to the eye peduncles (electronic supplementary material, figure S4a,c), suggesting that these tissues are not part of an eye peduncle, but belong to other structures (e.g. disassociated tissue from the head, antennae). Therefore, the eye peduncles are shorter than previously described. Annulated eye peduncles were previously identified in *Tuzoia* [25] and *Duplapex* [28]. In the former, the structure in question is probably not an eye peduncle: it appears close to the ventral side of the carapace, far from the cephalic region, and its length is close to 20% of the total length of the carapace, which would make it an extremely long structure compared to the eye peduncles known in other bivalved arthropods. In *Duplapex*, the potential eye peduncles do not bear eyes [28], making their affinity ambiguous. Instead, these structures are most probably compression artefacts on the carapace margins, similarly present in *Tuzoia* (Fig. 24;1 in [26]), which form as the lateral ridge compresses against the lateral margin of the carapace. Similar compression artefacts appear on the lateral edges of *Duplapex*, indicating that the full specimen was heavily dorsally compressed (Fig 4A–C in [28]), and have also been extensively reported in *Tuzoia* [33].

Two sclerotized structures are reported here for the first time. Several specimens (figures 1*d*, 3*e–h*; electronic supplementary material, figure S3c) show a rounded lobe between the peduncular lobe. A similar structure is present in several hymenocarines (e.g. *Odaraia*, *Perspicaris* and *Waptia* [44,46,47]), which is sometimes referred to as an anterior sclerite [44] or ocular tergite [5], and is also present in the closely related fuxianhuiids [48]. The anterior sclerite has been suggested to bear soft tissues underneath [5]. Following this hypothesis, the structure visible in *Tuzoia* would correspond to these soft tissues, given that all specimens available that are well-preserved show the head in ventral view. This would explain why the structure does not appear sclerotized or slightly differentiated from the remaining soft tissues on the head, as seen, for example, in *Perspicaris* [46]. The presence of a differentiated tergite, though, is difficult to infer from the material available here, and, for this reason, this trait was coded as unknown in both phylogenetic datasets. Another structure, a plate that appears sclerotized, is present in two specimens in ventral view (figures 2*a,b,c* and 3*e–h*). This structure is morphologically different from the putative anterior sclerite and resembles the hypostome of fuxianhuiids [48]. The fuxianhuiid hypostome is part of a complex system of sclerites and has also been interpreted as part of the labrum [14], but has not been observed in hymenocarines. For this reason, this character is considered dubitative and has not been coded into the phylogenetic matrix either (electronic supplementary material, *Discussion* 3.1).

Several structures have been previously interpreted as antennae in *Tuzoia* [25,26,45]*.* One specimen of *Tuzoia* shows an extremely thin, segmented structure originating close to the eye peduncle (electronic supplementary material, figure S4a,c), similar to the flagellate extensions of antennulae or antennae of some crustaceans [26]. A similar structure was also reported in *T. retifera*, but this appears poorly preserved [45]. This structure has not been observed in any other specimen here, and other limb-like appendages originating from the cephalon, which most probably represent antennae (figure 3*e,f*), are comparatively longer and thicker. It is possible that either this flagellate appendage does not belong to *Tuzoia*, or that due to its small size and thinness it is rarely preserved. The putative antenna described by Wen *et al.* [25] is largely triangular (Fig. 11A, B, E in [25]) and significantly thicker than the structure described in [26]. The morphology of this potential antenna is not clear from the published material, and it appears almost on the ventral side of the carapace, far from where the cephalon would be positioned. Based on its size and placement, then, its interpretation as an antenna is questionable. The specimens of *Tuzoia* showcased here show that the appendages are antenniform, but details on their annulation or exact morphology are unknown. For these reasons, this character is coded as present in the phylogenetic matrix, but other secondary related characters are considered unknown.

**Figure 8.**
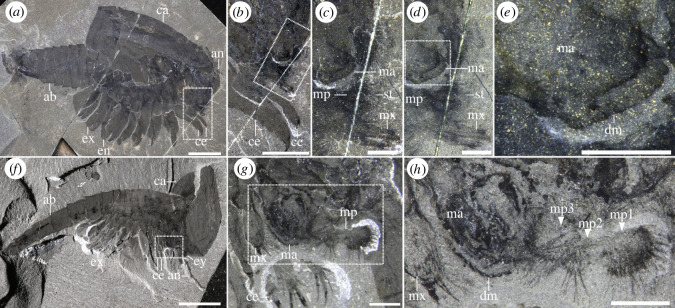
*Mandible and maxillulae in hymenocarines.* (*a*) *Canadaspis perfecta* (ROMIP61119) in lateral view, with legs exposed and multiple close-ups: post-antennular appendages and potential cephalothoracic legs (*b*), mandible with mandibular palp and maxillula in dry (*c*) and wet (*d*) conditions, mandible showing row of teeth (*e*); (*f*) *Waptia fieldensis* (ROMIP56432) in lateral view, with a close-up of the mandible and three-segmented mandibular palp under dry and wet conditions (*g,h*). Scales: (*a,f*) = 10 mm; (*b*) = 5 mm; (*c–e,g–h*) = 1 mm. Abbreviations: ab, abdomen; dm, dentate margin; ma, mandible; mx, maxilla; m.p, mandibular palp (and segments); st, setae.

Posterior to the antennae lies a potential differentiated cephalic appendage. This structure is three-segmented, short, and bears curved endites on each podomere (figure 4*e–g*). Similar structures are preserved in other bivalved arthropods including *Waptia* [12] and are here also illustrated for the first time in *Canadaspis* (figure 8), both of which can be confidently interpreted as mandibular palps. These structures are also different from the endites connected to the cephalothoracic legs, which are more elongated than round, and have strong spinose terminations (figure 4*e–g*). Despite the morphological similarities, mandibles are not visible in *Tuzoia*, and neither is a connection between these appendages and the head. Therefore, the identity of these structures as mandibular palps cannot be ascertained. For this reason, this and other related characters (e.g. mandibles) were coded as unknown in both phylogenetic datasets.

The presence of segmentation is not clear from the specimens available; without removal of the carapace, only specimens in ventral view would be able to show segmentation, but these specimens are poorly preserved (figure 1*a–c*; electronic supplementary material, figures S1 and S3). Specimens of *T. australis* show soft tissues belonging to the trunk [27] which may be subdivided into serially repeated units (see Plate 5 : 3,4 in [20]). These tissues are difficult to interpret, and serially repeated units could similarly correspond to the bases of the legs (as in figure 1*a–c,g*), instead. The number of legs in *Tuzoia* was obtained based on the size of the legs relative to the total size of the trunk and leg impressions in specimens in ventral view (figure 1*a–c*; electronic supplementary material figure S4f,g), leading to a total of 12 legs (two cephalothoracic), which could correspond approximately to the number of trunk subdivisions that can be observed in the specimens of *T. australis* [27]. Trunk segments were also reported in *T. tylodesa* (Fig. 12A-D in [25]): these are weakly preserved and extend posteriorly beyond the posterior edge of the carapace, similar to *Occacaris* [49]. The only specimen of *T. tylodesa* is *ca* 30 mm in length, small compared to the majority of specimens of *Tuzoia* (average length *ca* 70 mm, [26]), its carapace lacks a distinct anterior and posterior carapace process, and its trunk differs from that of *Tuzoia*, suggesting either that this is a juvenile stage in which relative size of the trunk compared to the carapace was larger in the initial growth stages of *Tuzoia*, or that *T. tylodesa* belongs to a different genus.

Trunk legs have also been previously reported from other sites [29,30], interpreted as ‘ventral appendages’ [30]. These are morphologically identical to the trunk legs shown herein, and thus, are most probably trunk legs displaced ventrally. A series of leg-like traces were also identified in *T. tylodesa* [25] preserved as faint red imprints, without clear morphological details. Two sets of legs are present in *Tuzoia*: cephalothoracic legs have a basipod with elongated endites (figure 4*c,e–g*) and bear spines in all podomeres (figure 4*d*). This contrasts with the morphology of the legs more posteriorly, which do not bear spines on all podomeres and their basipod lacks elongated endites (figures 3*h*, 4*c*, 5, 6*b*). The morphology of the trunk legs is constant across specimens, suggesting that the lack of endites and spines is not due to a different plane of orientation. Several legs show spines in both the second distalmost podomere and the basipod, reinforcing the idea that there is a differentiation between frontal (i.e. cephalothoracic) and posterior legs. This idea is also reinforced by the fact that both *Canadaspis* and *Waptia* have similar differentiated appendages, with their cephalothoracic legs bearing elongated endites that are not present in the remaining legs [12,50]. In *Waptia*, cephalothoracic legs are uniramous, and these could similarly be uniramous in *Canadaspis* (figure 8*b*) and are thus reconstructed as such in *Tuzoia* (figure 6). The basipod of *Tuzoia* is not entirely visible in the specimens available, but, when partly visible, does not show any evident sign of segmentation, strongly suggesting that this is a singular highly elongated structure (figures 4*c*, 5*c,f,g*), a differentiated, but not subdivided structure. The shape of the exopods in the present reconstruction was inferred from ellipsoid impressions (figure 1*a–c*, 2; electronic supplementary material, figure S1), but the exact morphology of these appendages is unknown. A series of filamentous setae related to the exopods had been previously reported in *Tuzoia* (Fig. 24: 1 in [26]). Re-examination of this material suggests that these are probably taphonomic artefacts present on the surface of the carapace, rather than distinct carbonaceous structures (electronic supplementary material, figure S4a).

The tail fan of *Tuzoia* bears two pairs of caudal rami, observed in two specimens (figure 1*a,c,e,f, i–k*; electronic supplementary material, figure S2). The composite of part and counterpart shows that each structure belongs to a different fossil layer (figure 1*k*). Given that segmentation is not visible among our specimens, it is unclear whether both pairs of caudal rami originate from the same terminal segment or each from a different one. One pair of thin, highly elongated posterior appendages has been previously described in *T. parva* [22,46]*,* not observed here. The affinities of *T*. *parva* are contentious, and similar to *T. tylodesa*, could represent a larval stage of *Tuzoia* or a different genus altogether [22,46].

### 4.2. Phylogenetic affinities of *Tuzoia*

**Figure 9.**
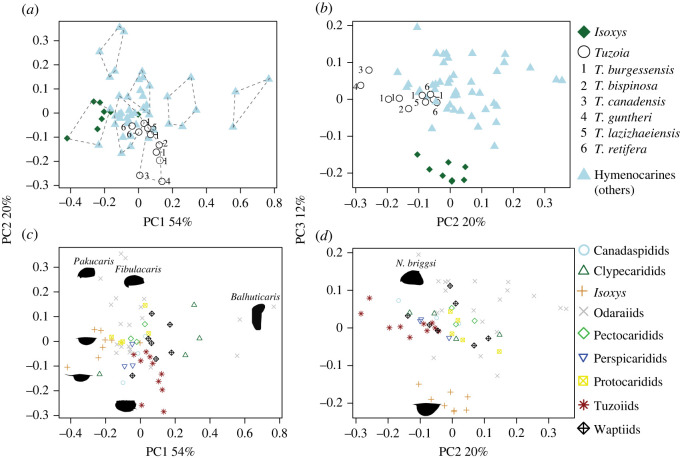
*Carapace morphospace results.* Results from the morphospace containing 63 shapes for a total of 20 genera of bivalved arthropods. Dataset differentiating hymenocarines (including *Tuzoia*) from isoxyids (*a,b*). Results of the K-means clustering results (seven clusters) shown on the first and second component (*a*). Isoxyids appear differentiated from hymenocarines in the first and third component and most of its specimens are clustered together. *Tuzoia* appears closer to hymenocarines, either in its own cluster or with other hymenocarines. Dataset differentiating hymenocarine groups (*c,d*), including: canadaspidids (*Canadaspis*), clypecaridids (*Clypecaris*, *Ercaicunia*, *Plenocaris*), odaraiids (*Balhuticaris*, *Fibulacaris*, *Jugatacaris*, *Nereocaris*, *Odaraia*, *Pakucaris*, *Vermontcaris*), pectocaridids (*Pectocaris*), perspicaridids (*Perspicaris*), protocaridids (*Branchiocaris*, *Tokummia*), tuzoiids (*Tuzoia*) and waptiids (*Pauloterminus*, *Waptia*). Odaraiids comprise most of the occupied morphospace, and extreme morphologies (e.g. *Fibulacaris*, *Balhuticaris*) carry much of the weight of the component. Silhouettes represent the shapes at the extremes of the components.

It has been previously suggested that *Tuzoia* may be closely related to the bivalved stem-euarthropod *Isoxys* (Isoxyidae) based on the shared presence of reticulation on the carapace, dorsal anterior and posterior carapace processes and the presence of large eyes [25,26,28,51]. Both genera also experienced similar preservation biases and were consequently inferred to be pelagic, thus leading to further non-phylogenetic comparisons [27,52]. Carapace morphology was also used to propose a close relation to the potential hymenocarine [16] *Pseudoarctolepis* [51] and crustacean thylacocephalans [53], similarly based on the shared presence of anterior and posterior dorsal carapace processes. Several of these features, though, are prone to appear convergently: reticulate patterns are not exclusive to *Tuzoia* and *Isoxys* and can be found across other hymenocarines [46,54] as well as in other arthropods [55]. Similarly, carapace processes are a common adaptation to predatory deterrence and hydrodynamic streamlining [52], and are also present in several other hymenocarines, as well [5,56]. Whether the shape of the carapace of *Tuzoia* closely resembles that of isoxyids is tested here through a carapace morphospace. The morphospace is able to separate *Isoxys* from hymenocarines on the first and third components, and isoxyids appear as a differentiated cluster when the dataset is divided into more than two clusters (electronic supplementary material, *Analyses 2.1*). *Tuzoia* clusters with other hymenocarines, and its different species are grouped as a distinct cluster when more than five subdivisions of the dataset are chosen (figure 9). This differentiation is further endorsed by a previously published carapace morphospace [52] that separated *Tuzoia* and isoxyids in the second component and differentiates them into separate clusters.

Isoxyids are characterized by the presence of a pair of strong subchelate appendages of variable morphology [57,58]: these are generally large compared to the body length, thick, usually subdivided into few podomeres, and bear distinct spines on their ventral side (and dorsal side in *Surusicaris* [57]). Spines can be located on the medio-ventral margin of each podomere (in *I. acutangulus*) [21], sometimes with adjacent smaller ones [58] (in *I. curvirostratus*). In *I. auritus*, appendages are highly elongated and multi-podomerous and could even be classified as antenniform [21]. *Tuzoia* bears a pair of antenniform appendages (i.e. the antennae) that do not resemble the frontalmost appendages in isoxyids; these are short, but not thick, and have no evidence of being subdivided into a few large spinose podomeres. These appendages are therefore more similar to a euarthropod antenna. On the other hand, it is also worth noting the variability across isoxyid appendages, especially considering *I. auritus*, and how spinose antennae can also be found in some hymenocarines (*Clypecaris* and *Plenocaris* [59,60]).

**Figure 10.**
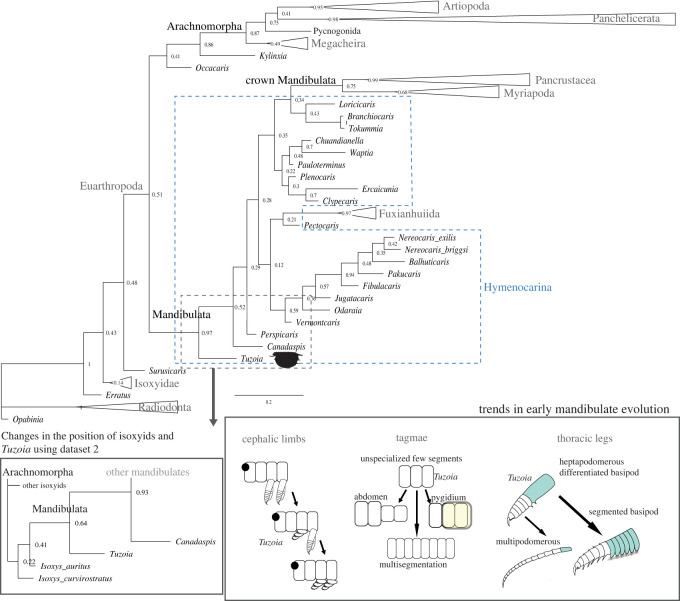
*Phylogenetic analysis and trends in early mandibulate evolution*. (*a*) Simplified version of the results of the phylogenetic analysis dataset 1, showcasing the position of *Tuzoia* within a paraphyletic Hymenocarina as an early mandibulate group. The results of the more conservative dataset (dataset 2) are shown as an inset containing only the closest allied taxa to *Tuzoia* (see electronic supplementary material, figure S6 for full tree). The diagrams (right) represent several hypothetical trends across early mandibulate evolution and the potential position of *Tuzoia* within these transitions. These include the reduction and integration of thoracic legs into a head specialized in feeding, the increasing diversity of post-cephalic tagmae and changes in leg morphology from a heptopodomerous leg with differentiated basipod.

Further similarities between *Tuzoia* and hymenocarines (e.g. *Waptia*) include a pair of peduncular lobes and a singular potential sclerite present between the eyes [12]. These are non-appendicular structures found across multiple euarthropods but have not been observed in isoxyids, so far [61]. The head of *Tuzoia* is also specialized: it bears one pair of differentiated cephalic appendages (potential mandibular palps) and at least two pairs of differentiated cephalothoracic legs. Based on their position as stem-euarthropods, isoxyids have sometimes been reconstructed as lacking differentiation on their frontal thoracic legs [19,21], but more recent studies show that the frontal legs may be differentiated into a more specialized head [57,62]. Following this interpretation, it could be argued that the cephalothoracic legs of *Tuzoia* could also belong to a specialized isoxyid head. Instead, these legs are here homologized to the cephalothoracic legs in hymenocarines based on: the potential presence of an antenna and a differentiated cephalic appendage, and clear euarthropod traits on the legs, including a strong arthropodization [63], an heptopodomerous endopod [64] and a differentiated basipod [65]. By contrast, isoxyid legs are usually poorly segmented and sclerotized [57], have multi-podomerous endopods [62], and their basipod is considered undifferentiated under several different evolutionary scenarios [65,66]. Dataset 2 was coded to consider the head of *Tuzoia* homologous to that in isoxyids, but this did not change the results of the phylogenetic analysis (figure 10). The presence of an enlarged unsegmented basipod together with an heptopodomerous endopod is though also unique among hymenocarines.

Similarly, the tagmatization of *Tuzoia* is also unique among hymenocarines, and more closely resembles that of isoxyids: the trunk bears a limited number of legs (*ca* 10), lacks an abdomen and is almost entirely covered by the carapace. This contrasts with the highly multi-segmented homonomous trunk of ‘odaraiids’ and protocaridids [5,67], but also with the trunk of canadaspidids or waptiids, which bear a limbless abdomen that protrudes extensively beyond the carapace. Most hymenocarines also bear a single pair of caudal rami (see [4]), with each caudal ramus being independent (although partly fused in *Jugatacaris* [68]), but *Tuzoia* has two pairs of fused caudal rami. This conformation is also reminiscent to the tail fans present in isoxyids and *Erratus* with multiple tail flukes and the posteriormost pair being fused into a singular truncate paddle [21,69].

Our phylogenetic analysis supports a 'deep-split’ scenario [70] using both datasets (figure 10). In this scenario, the total group Mandibulata and the remaining arthropod groups originate soon after the origin of the first euarthropods [13]. This result contrasts with previous phylogenetic analyses which indicated an early branching of megacheirans, artiopodans and panchelicerates, followed by a later origin of the mandibulate total group [12,14,58], as well as analyses that placed hymenocarines as the earliest euarthropods [54,65]. The results of the phylogenetic analysis show that *Tuzoia* most probably belongs to a paraphyletic Hymenocarina, including the non-bivalved fuxianhuiids, all resolved as early mandibulates (figure 10; electronic supplementary material, figure S5), with multiple synapomorphic characters shared with hymenocarines and several others potential convergent with isoxyids (electronic supplementary material, *Discussion 3.2*).

*Tuzoia* appears as the basalmost hymenocarine, but given the low-phylogenetic support across hymenocarine subgroups, this specific position remains tentative. The deep-split scenario and position of *Tuzoia* within Mandibulata are also present in the more conservative phylogenetic dataset (dataset 2), which otherwise shows a paraphyletic Isoxyidae (electronic supplementary material, figure S6). The position of *Tuzoia* as an early mandibulate is present in the majority of trees in both datasets, based on the treespace visualization (electronic supplementary material, figures S5 and S6).

### 4.3. Implications in early mandibulate evolution

Finding mandibulate synapomorphies represents a contentious issue; two of the three main mandibulate groups (myriapods and hexapods) are terrestrial, and thus, their morphology is highly derived from a marine mandibulate ancestor. Hymenocarines may represent a candidate for a mandibulate stem-group, but their morphological disparity [13] is becoming increasingly higher as their diversity increases [37,71], further impeding a clear scenario for the early evolution of mandibulates. The results of the phylogenetic analysis suggest that the origin of the total group Mandibulata could have been characterized by a fast radiation of different disparate body plans soon after the origin of euarthropods. It has been hypothesized that hymenocarines are early euarthropods with convergent mandibulate traits [11], but our results support hymenocarines as early mandibulates that could have retained more plesiomorphic characters, instead. *Tuzoia* is recovered as an hymenocarine, but its body plan suggests that several isoxyid traits were retained from the stem-euarthropod ancestor or, alternatively, appeared convergently.

One of the evolutionary trends of mandibulates is the reduction of thoracic appendages (figure 10), their specialization and their integration into the head as they acquire a feeding function [5,72]. The differentiated post-antennular appendage of *Tuzoia* and cephalothoracic legs illustrate this trend. If *Tuzoia*, supported by its isoxyid-like characters, is a basal hymenocarine, this would imply that these specializations occurred early in the evolution of mandibulates and could be used to define and determine the origin of Mandibulata. This is not surprising if we consider fuxianhuiids, in which the specialized post-antennular appendage is reduced and had a feeding function, and the following five tergites and legs were similarly reduced in size [14]. The head of the ‘odaraiid’ hymenocarines remains unresolved, but several species already indicate certain post-antennular specializations, such as the third cephalic appendages of *Pakucaris* [71] or the thorax of *Balhuticaris*, in which the legs become increasingly smaller towards the head and are part of a differentiated unit from the trunk [37]. This specialization and integration into the head are best seen across hymenocarine taxa with mandibles and one pair of maxillae such as *Canadaspis* (figure 8*a–e*), *Waptia* (figure 8*f–h*) or *Ercaicunia* [4]. The presence of a mandible has been long recognized as a mandibulate autapomorphy [72–74]. The developmental stages of pancrustacean larvae like *Rehbachiella* [75] show that the basalmost part of the leg (the proximal endite or coxa) becomes enlarged and gnathobasic as the remaining parts of the legs become reduced and a similar process could have probably occurred at the early stages of mandibulate evolution. The morphological changes from a thoracic leg to a mandible are currently not present in the hymenocarine fossil record, and the lack of clear cephalic conformations for many of its taxa impedes a full reconstruction of this evolutionary transition, though.

*Tuzoia*'s trunk is short, with few segments, unspecialized and mostly enclosed within the carapace. This could represent a potential plesiomorphic trait carried from isoxyids or a convergent trait related to a similar lifestyle. It is important to note that tagmatization appears to be highly disparate among hymenocarines [37], even within subgroups with strong monophyletic support [71], suggesting that this was a highly plastic trait in early mandibulate evolution. Another unique trait of *Tuzoia* compared to other hymenocarines and fuxianhuiids is its differentiated, highly elongated, unsegmented basipod. A differentiated basipod has been proposed as a synapomorphy uniting mandibulates and arachnomorphs under the hypothesis of hymenocarines being mandibulates [66] but also as an apomorphy that would only appear in fuxianhuiids, mandibulates and arachnomorphs if hymenocarines would represent early euarthropods [63–66]. Regardless of different competing scenarios, if *Tuzoia* was an isoxyid, this would indicate that full arthropodization of the legs, including a strong sclerotization, reduction of the endopod into seven podomeres and differentiation of the basipod, all occurred before the origin of euarthropods. On the other hand, the position of *Tuzoia* as a mandibulate best aligns with previously hypothesized evolutionary scenarios for this group. It has been suggested that the ancestral mandibulate leg had a highly elongated basipod with enditic lobes along its inner margin, similar to the Cambrian pancrustacean *Rehbachiella* [72]. The basipod would later become segmented, differentiating into the coxa and precoxa of extant mandibulates, evinced by the proximal endite of *Rehbachiella* [72]. *Tuzoia* could represent the actual ancestral mandibulate leg condition, with an elongated basipod either with (in its cephalothoracic legs) or without (in its trunk legs) developed enditic lobes. The basipod would later become further specialized (e.g. fuxianhuiids [65]) or multi-segmented (e.g. *Waptia* [12]) and would finally become constrained into one or two subdivisions (e.g. *Ercaicunia* [4]). This scenario also implies that the ancestral mandibulate leg could have had an heptopodomerous endopod, a character that could also be present in the euarthropod common ancestor [66]. The multi-podomerous endopods observed in fuxianhuiids [65] and ‘odaraiids’ [71], then, would be the result of a secondary subdivision, rather than representing a more ancestral trait, as previously hypothesized [65].

Stem-euarthropods such as opabiniids [76], several radiodonts (e.g. *Anomalocaris* [77]) and some isoxyids [21,69] are characterized by a tail fan composed of multiple pairs of blade-like tail flukes. This condition is not present among euarthropods but it is worth noting that radiodonts were not restricted to this specific condition either, and show widely different types of tail flukes (e.g. *Hurdia* [78] and *Schinderhannes* [79]). This type of tail fan was originally homologized to the caudal rami of the hymenocarine *Nereocaris exilis*, which had been interpreted as several pairs of tail flukes and helped support the position of hymenocarines as early euarthropods [9]. These, however, are probably a pair of tripartite caudal rami, instead, and thus, a different structure altogether [37]. The tail fan of *Tuzoia* and its resemblance to that of isoxyids poses an interesting question. Arthropodization in stem-euarthropods radiodonts was restricted to the frontalmost pair of legs [80], implying that if the tail of *Tuzoia* is homologous, the tail flukes are not arthropodized. The retention of ancestral features can lead to non-parsimonious character transitions, but cases such as the lobopodous legs of *Surusicaris* [57] or the frontal radiodont-like appendages of *Kylinxia* [58] suggest that these retentions could have occurred under a scenario with low morphological constraints and low canalization. If that is the case, it is possible that tail flukes were still present within euarthropods, but were experimenting with a progressive reduction, as shown by the decreasing number of tail flukes from opabiniids and radiodonts [76] to isoxyids [21] and to early mandibulates (*Tuzoia*), as sclerotized structures (exopods and caudal rami) replaced their swimming function. A potential alternative to this scenario is that the tail fan of *Tuzoia* is not homologous to that of isoxyids, but a convergent structure. The tail fan could represent either two pairs of true caudal rami or one pair of caudal rami together with an extended telson.

### 4.4. Palaeoecology

Previous interpretations of the lifestyle of *Tuzoia* have been based on patterns of preservation, the shape of the carapace, its eye size [18] and wide distribution, all potentially indicative of a pelagic lifestyle [26]. *Tuzoia* carapaces are abundant at the Burgess Shale [26] and other open marine environments (e.g. Kaili Biota [82,83]), but other soft tissues in these sites are extremely rare. It has been suggested, then, that most specimens in these sites represent moults or carcasses [84] falling from a pelagic community [26]. The taphonomic bias against soft tissues present at the Burgess Shale may not be shared across Burgess Shale-type deposits. In some deposits (e.g. Emu Bay), the proportion of specimens of *Tuzoia* with soft tissues (albeit poorly preserved) seems higher, despite a relatively low total number of specimens [27], which could be related to the higher number of preserved pelagic taxa in this locality [85]. On the other hand, not only the position in the water column, but also size can play a role in preservation; large swimming species tend to be rare in Burgess Shale-type deposits and are represented mostly by carcasses or isolated appendages [55] seemingly because such animals would have been able to escape burial events more easily. Carapace reticulation can occur irrespective of the lifestyle of the animal [26], and carapace processes, while having important hydrodynamical roles [86], can also have defensive roles [87], and are not restricted to pelagic inhabitants [67]. Large eyes can be indicative of a wide range of vision and light reception, suitable for a pelagic lifestyle [19], but in *Tuzoia* these are not placed fronto-ventrally but are rather facing frontally, a condition closer to the benthic *Waptia* [12]. The worldwide distribution of *Tuzoia* is not necessarily explained by a pelagic lifestyle [26], either. The benthic *Naraoia* was also one of the most widely distributed genera of the Cambrian [24], and widespread distributions can be easily achieved through larval, planktonic stages [88].

The traits listed previously: taphonomic bias, reticulation, eye size and distribution, then, do not necessarily imply a strict pelagic lifestyle. The legs of *Tuzoia* terminate in distinct claw-shaped podomeres. These terminations are similarly present in extant pancrustaceans that walk on the substrate or anchor themselves to hard structures (e.g. amphipods, [89]; atyid prawns, [90]; slipper lobsters [91]). Given that the legs are shorter than the height of the carapace valve, though, the carapace valves must have been able to partially open, allowing the legs to reach the substrate. The carapace of *Tuzoia* presents a medial hinge, indicative that the frontoposterior axis was weakly sclerotized, allowing the valves to open. After moulting, carapaces with hinge lines would be prone to split, resulting in isolated valves, abundant in this and other bivalved arthropods of the Burgess Shale [92].

The cephalothoracic frontalmost endopods are stout and bear strong spines, indicative of a predatory or scavenging function, as previously suggested [93]. These legs do not extend widely beyond the body, suggesting that they were probably not suited to be used as a raptorial appendage, capturing prey from afar. The distal claw-shaped podomere and adjacent podomere with elongated spines of *Tuzoia* could have been used similar to the pereopods in benthic slipper lobsters [94], which are used to capture sessile, slow-moving prey or handle carcasses in the proximity. These legs are also reminiscent of the maxillipeds in several crustacean taxa [95,96], which have a primarily locomotory function but can also handle prey or tissues and bring them to the mouthparts [97]. Despite this, the paddle-shaped exopods, the elongated basipod [72] and the large tail fan are indicative that *Tuzoia* was also an active swimmer. Benthic extant decapods such as spiny and slipper lobsters [98,99] have similarly wide tail fans, mostly used for escape reactions, but also make use of their elongated pleon in the process, while the trunk of *Tuzoia* is comparatively shorter*.* The Cambrian radiodont *Anomalocaris* has a similarly large triangular tail compared to a comparatively short body and its tail may have assisted in turning manoeuvres [100]. The tail of *Tuzoia* could have had a similar function and the lateral ridges of the carapace could have further assisted in buoyancy and directionality. Altogether, *Tuzoia* was probably an active swimmer, but not restricted to a pelagic lifestyle, having instead a broader nektobenthic predatory or scavenging ecology.

## 5. Conclusion

The new reconstruction of *Tuzoia* makes use of extensive information provided by soft tissues to resolve a 100-year-old question regarding the affinities and ecology of this animal. *Tuzoia* had a nektobenthic to pelagic predatory or scavenging role, completing an information gap across multiple Cambrian communities. Previous comparisons to the stem-euarthropod *Isoxys* are refuted, arguing for a position among early mandibulates, instead. *Tuzoia* illustrates some of the evolutionary trends towards the origin of mandibulates: the recruitment of thoracic legs into the head through reduction and specialization, the diversification of trunk tagma and the differentiation of the basipod. On the other hand, other traits are closer to those in isoxyids, either indicating a case of convergent evolution or the potential retention of traits from the stem-euarthropod group. Key to this is the position of *Tuzoia* within hymenocarines, which remains highly hypothetical: hymenocarines are an increasingly disparate group with limited information on key diagnostic areas. Their phylogeny, and even their position in early arthropod evolution, thus remains a highly debated topic [4,101]. The discovery of more soft tissues and a full comprehension of the anatomical diversity of this group is then the first essential step to tackling this debate.

## Data Availability

All supporting materials can be found in the electronic supplementary material [102].

## References

[1] Rota-Stabelli O et al. 2011 A congruent solution to arthropod phylogeny: phylogenomics, microRNAs and morphology support monophyletic Mandibulata. R. Soc. B Biol. Sci. **278**, 298-306. (10.1098/rspb.2010.0590) PMC3013382 20702459

[2] Wolfe JM, Daley AC, Legg DA, Edgecombe GD. 2016 Fossil calibrations for the arthropod Tree of Life. Earth Sci. Rev. **160**, 43-110. (10.1016/j.earscirev.2016.06.008)

[3] Wolfe JM. 2017 Metamorphosis is ancestral for crown euarthropods, and evolved in the Cambrian or earlier. Integr. Comp. Biol. **57**, 499-509. (10.1093/icb/icx039) 28957514

[4] Zhai D, Ortega-Hernández J, Wolfe JM, Hou XG, Cao C, Liu Y. 2019 Three-dimensionally preserved appendages in an early Cambrian stem-group pancrustacean. Curr. Biol. **29**, 171-177. (10.1016/j.cub.2018.11.060) 30595518

[5] Aria C, Caron JB. 2017 Burgess Shale fossils illustrate the origin of the mandibulate body plan. Nature **545**, 89-92. (10.1038/nature22080) 28445464

[6] Giribet G, Edgecombe GD. 2019 The phylogeny and evolutionary history of arthropods. Curr. Biol. **29**, R592-R602. (10.1016/j.cub.2019.04.057) 31211983

[7] Legg D, Sutton MD, Edgecombe GD. 2013 Arthropod fossil data increase congruence of morphological and molecular phylogenies. Nat. Commun. **4**, 1-7. (10.1038/ncomms3485) 24077329

[8] Wolfe JM, Hegna TA. 2014 Testing the phylogenetic position of *Cambrian pancrustacean* larval fossils by coding ontogenetic stages. Cladistics **30**, 366-390. (10.1111/cla.12051) 34788971

[9] Legg DA, Sutton MD, Edgecombe GD, Caron JB. 2012 Cambrian bivalved arthropod reveals origin of arthrodization. Proc. R. Soc. B **279**, 4699-4704. (10.1098/rspb.2012.1958) PMC3497099 23055069

[10] Ortega-Hernández J, Janssen R, Budd GE. 2017 Origin and evolution of the panarthropod head – A palaeobiological and developmental perspective. Arthropod. Struct. Dev. **46**, 354-379. (10.1016/j.asd.2016.10.011) 27989966

[11] Budd GE. 2021 The origin and evolution of the euarthropod labrum. Arthropod. Struct. Dev. **62**, 101048. (10.1016/j.asd.2021.101048) 33862532

[12] Vannier J, Aria C, Taylor RS, Caron JB. 2018 *Waptia fieldensis* Walcott, a mandibulate arthropod from the middle Cambrian Burgess Shale. R. Soc. Open Sci. **5**, 172206. (10.1098/rsos.172206) 30110460 PMC6030330

[13] Aria C. 2020 Macroevolutionary patterns of body plan canalization in euarthropods. Paleobiology **46**, 569-593. (10.1017/pab.2020.36)

[14] Aria C, Zhao F, Zhu M. 2021 Fuxianhuiids are mandibulates and share affinities with total-group Myriapoda. J. Geol. Soc. Lond. **178**, jgs2020-246. (10.1144/jgs2020-246)

[15] Yuan J, Peng J, Zhao Y. 2011 New bivalved arthropods from Mid-Cambrian Kaili Biota of Southeastern Guizhou, Southwest China. Acta Geol. Sin English Ed. **85**, 758-764. (10.1111/j.1755-6724.2011.00481.x)

[16] Lerosey-Aubril R, Kimmig J, Pates S, Skabelund J, Weug A, Ortega-Hernández J. 2020 New exceptionally preserved panarthropods from the Drumian Wheeler Konservat-Lagerstätte of the House Range of Utah. Pap. Palaeontol. **6**, 501-531. (10.1002/spp2.1307)

[17] Walcott CD. 1890 The fauna of the Lower Cambrian or Olenellus Zone. *Tenth annual report of the director, 1888–1889. Part 1.* Reston, VA: United States Geological Survey.

[18] Vannier J, Chen J. 2000 The Early Cambrian colonization of pelagic niches exemplified by *Isoxys* (Arthropoda). Lethaia **33**, 295-311. (10.1080/002411600750053862)

[19] Vannier J, García-Bellido DC, Hu SX, Chen AL. 2009 Arthropod visual predators in the early pelagic ecosystem: evidence from the Burgess Shale and Chengjiang biotas. Proc. R. Soc. B **276**, 2567-2574. (10.1098/rspb.2009.0361) PMC2686666 19403536

[20] García-Bellido DC, Vannier J, Collins D. 2009 Soft-part preservation in two species of the arthropod *Isoxys* from the middle Cambrian Burgess Shale of British Columbia, Canada. Acta Palaeontol. Pol. **54**, 699-712. (10.4202/app.2009.0024)

[21] Legg DA, Vannier J. 2013 The affinities of the cosmopolitan arthropod *Isoxys* and its implications for the origin of arthropods. Lethaia **46**, 540-550. (10.1111/let.12032)

[22] Walcott CD. 1912 Cambrian geology and paleontology II: Middle Cambrian Branchiopoda, Malacostraca, Trilobita and Merostomata. Smithson Misc. Collect. **57**, 145-228. (10.1017/S0016756800115006)

[23] Resser CE. 1929 New Lower and Middle Cambrian Crustacea. Proc. US Natl Mus. **76**, 1-18. (10.5479/si.00963801.76-2806.1)

[24] Hendricks JR, Lieberman BS, Stigall AL. 2008 Using GIS to study palaeobiogeographic and macroevolutionary patterns in soft-bodied Cambrian arthropods. Palaeogeogr. Palaeoclimatol. Palaeoecol. **264**, 163-175. (10.1016/j.palaeo.2008.04.014)

[25] Wen R, Babcock LE, Peng J, Liu S, Liang B. 2019 The bivalved arthropod *Tuzoia* from the Balang Formation (Cambrian Stage 4) of Guizhou, China, and new observations on comparative species. Pap. Palaeontol. **5**, 719-742. (10.1002/spp2.1262)

[26] Vannier J et al. 2007 *Tuzoia*: morphology and lifestyle of a large bivalved arthropod of the Cambrian seas. J. Paleontol. **81**, 445-471. (10.1666/05070.1)

[27] García-Bellido DC, Paterson JR, Edgecombe GD, Jago JB, Gehling JG, Lee MSY. 2009 The bivalved arthropods *Isoxys* and *Tuzoia* with soft-part preservation from the lower Cambrian Emu Bay Shale lagerstätte (Kangaroo Island, Australia). Palaeontology **52**, 1221-1241. (10.1111/j.1475-4983.2009.00914.x)

[28] Ma J et al. 2021 A new bivalved arthropod from Cambrian (Stage 3) Qingjiang biota expands the palaeogeographical distribution and increases the diversity of Tuzoiidae. J. Geol. Soc. Lond. **179**, 229. (10.1144/jgs2020-229)

[29] Caron JB, Gaines RR, Mángano MG, Streng M, Daley AC. 2010 A new Burgess Shale-type assemblage from the ‘thin’ Stephen Formation of the southern Canadian Rockies. Geology **38**, 811-814. (10.1130/G31080.1)

[30] Du KS et al. 2020 A new early Cambrian Konservat-Lagerstätte expands the occurrence of Burgess Shale-type deposits on the Yangtze Platform. Earth-Sci. Rev. **211**, 103409. (10.1016/j.earscirev.2020.103409)

[31] Chen WY, Zhao YL, Yang XL, Wen RQ. 2017 *Tuzoia* Walcott, 1912 from the Cambrian ‘Tsinghsutung Formation’ of Guizhou, China. Palaeontol. Sin. **56**, 301-311. (10.19800/j.cnki.aps.2017.03.004)

[32] Gaines RR, Briggs DEG, Zhao Y. 2008 Cambrian Burgess Shale-type deposits share a common mode of fossilization. Geology **36**, 755-758. (10.1130/G24961A.1)

[33] Mángano MG, Hawkes CD, Caron JB. 2019 Trace fossils associated with Burgess Shale non-biomineralized carapaces: bringing taphonomic and ecological controls into focus. R. Soc. Open Sci. **6**, 172074. (10.1098/rsos.172074) 30800334 PMC6366168

[34] Bonhomme V, Picq S, Gaucherel C, Claude J. 2014 Momocs: outline analysis using R. J. Stat. Softw. **56**, 1-24. (10.18637/jss.v056.i13)

[35] Adams DC, Otárola-Castillo E. 2012 geomorph: an r package for the collection and analysis of geometric morphometric shape data. Methods Ecol. Evol. **4**, 393-399. (10.1111/2041-210X.12035)

[36] Aria C, Zhao F, Zeng H, Guo J, Zhu M. 2020 Fossils from South China redefine the ancestral euarthropod body plan. BMC Evol. Biol. **20**, 1-17. (10.1186/s12862-019-1560-7) 31914921 PMC6950928

[37] Izquierdo-López A, Caron JB. 2022 Extreme multisegmentation in a giant bivalved arthropod from the Cambrian Burgess Shale. iScience **25**, 104675. (10.1016/j.isci.2022.104675) 35845166 PMC9283658

[38] Moysiuk J, Caron JB. 2022 A three-eyed radiodont with fossilized neuroanatomy informs the origin of the arthropod head and segmentation. Curr. Biol. **32**, 1-15. (10.1016/j.cub.2022.06.027) 35809569

[39] Ronquist F et al. 2012 MrBayes 3.2: efficient Bayesian phylogenetic inference and model choice across a large model space. Syst. Biol. **61**, 539-542. (10.1093/sysbio/sys029) 22357727 PMC3329765

[40] Rambaut A, Drummond AJ, Xie W, Baele G, Suchard MA. 2018 Posterior summarisation in Bayesian phylogenetics using Tracer 1.7. Syst. Biol. **67**, 901-904. (10.1093/sysbio/syy032) 29718447 PMC6101584

[41] Paradis E, Schliep K. 2019 ape 5.0: an environment for modern phylogenetics and evolutionary analyses in R. Bioinformatics **35**, 526-528. (10.1093/bioinformatics/bty633) 30016406

[42] Schliep KP. 2011 phangorn: phylogenetic analysis in R. Bioinformatics **27**, 592-593. (10.1093/bioinformatics/btq706) 21169378 PMC3035803

[43] Clarke JM, Ruedemann R. 1912 The Eurypterida of New York. *New York State Museum, Memoir Vol. 14.* New York, NY: New York State Museum.

[44] Budd GE. 2008 Head structure in upper stem-group euarthropods. Palaeontology **51**, 561-573. (10.1111/j.1475-4983.2008.00752.x)

[45] Wu Y, Liu J. 2022 New data on the bivalved arthropod *Tuzoia* from the Cambrian (Series 2, Stage 4) Guanshan Biota in Kunming, Yunnan, Southwest China. Front. Earch Sci. **10**, 862679. (10.3389/feart.2022.862679)

[46] Briggs DEG. 1977 Bivalved arthropods from the Cambrian Burgess Shale of British Columbia. Palaeontology **20**, 595-621.

[47] Strausfeld NJ. 2016 *Waptia* revisited: intimations of behaviors. Arthropod. Struct. Dev. **45**, 173-184. (10.1016/j.asd.2015.09.001) 26365952

[48] Yang J, Ortega-Hernández J, Butterfield NJ, Zhang XG. 2013 Specialized appendages in fuxianhuiids and the head organization of early euarthropods. Nature **494**, 468-471. (10.1038/nature11874) 23446418

[49] Hou XG. 1999 New rare bivalved arthropods from the lower Cambrian Chengjiang fauna, Yunnan, China. J. Paleontol. **73**, 102-116. (10.1017/S002233600002758X)

[50] Briggs DEG. 1978 The morphology, mode of life, and affinities of *Canadaspis perfecta* (Crustacea: Phyllocarida), middle Cambrian, Burgess Shale, British Columbia. Phylosophical Trans. R. Soc. Lond. **281**, 439-487. (10.1098/rstb.1978.0005)

[51] Williams M, Siveter DJ, Peel JS. 1996 *Isoxys* (Arthropoda) from the Early Cambrian Sirius Passet Lagerstätte, North Greenland. J. Paleontol. **70**, 947-954. (10.1017/S0022336000038646)

[52] Pates S, Daley AC, Legg DA, Rahman IA. 2021 Vertically migrating *Isoxys* and the early Cambrian biological pump. Proc. R. Soc. B **288**, 20210464. (10.1098/rspb.2021.0464) PMC8220267 34157876

[53] Vannier J, Chen JY, Huang DY, Charbonnier S, Wang XQ. 2006 The Early Cambrian origin of thylacocephalan arthropods. Acta Palaeontol. Pol. **51**, 201-214.

[54] Legg DA, Caron JB. 2014 New Middle Cambrian bivalved arthropods from the Burgess Shale (British Columbia, Canada). Palaeontology **57**, 691-711. (10.1111/pala.12081)

[55] Moysiuk J, Caron JB. 2019 A new hurdiid radiodont from the Burgess Shale evinces the exploitation of Cambrian infaunal food sources. Proc. R. Soc. B **286**, 20191079. (10.1098/rspb.2019.1079) PMC6710600 31362637

[56] Briggs DEG. 1976 The arthropod *Branchiocaris* n. gen., middle Cambrian, Burgess Shale, British Columbia. Geol. Surv. Canada Energy Mines Resour. Canada **264**, 1-29.

[57] Aria C, Caron JB. 2015 Cephalic and limb anatomy of a new isoxyid from the Burgess Shale and the role of ‘stem bivalved arthropods' in the disparity of the frontalmost appendage. PLoS ONE **10**, 1-37. (10.1371/journal.pone.0124979) PMC4454494 26038846

[58] Zeng H, Zhao F, Niu K, Zhu M, Huang D. 2020 An early Cambrian euarthropod with radiodont-like raptorial appendages. Nature **588**, 101-105. (10.1038/s41586-020-2883-7) 33149303

[59] Yang J, Ortega-Hernández J, Lan T, Hou JB, Zhang XG. 2016 A predatory bivalved euarthropod from the Cambrian (Stage 3) Xiaoshiba Lagerstätte, South China. Sci. Rep. **6**, 27709. (10.1038/srep27709) 27283406 PMC4901283

[60] Whittington HB. 1974 *Yohoia* Walcott and *Plenocaris* n. gen., arthropods from the Burgess Shale, Middle Cambrian, British Columbia. Geol. Surv. Canada Bull. **231**, 1-63. (10.1017/S0016756800045921)

[61] Ortega-Hernández J, Budd GE. 2016 The nature of non-appendicular anterior paired projections in Palaeozoic total-group Euarthropoda. Arthropod. Struct. Dev. **45**, 185-199. (10.1016/j.asd.2016.01.006) 26802876

[62] Zhang C et al. 2021 Differentiated appendages in *Isoxys* illuminate origin of arthropodization (preprint, 2021). Researchsquare. (10.21203/rs.3.rs-861892/v1)

[63] Ortega-Hernández J. 2014 Making sense of ‘lower’ and ‘upper’ stem-group Euarthropoda, with comments on the strict use of the name Arthropoda von Siebold, 1848. Biol. Rev. **91**, 255-273. (10.1111/brv.12168) 25528950

[64] Aria C, Caron JB. 2019 A middle Cambrian arthropod with chelicerae and proto-book gills. Nature **573**, 586-589. (10.1038/s41586-019-1525-4) 31511691

[65] Yang J, Ortega-Hernández J, Legg DA, Lan T, Hou JB, Zhang XG. 2018 Early Cambrian fuxianhuiids from China reveal origin of the gnathobasic protopodite in euarthropods. Nat. Commun. **9**, 1-9. (10.1038/s41467-017-02754-z) 29391458 PMC5794847

[66] Aria C. Reviewing the bases for a nomenclatural uniformization of the highest taxonomic levels in arthropods. Geol. Mag. 2019;**156**, 1463-1468. 10.1017/S0016756819000475

[67] Izquierdo-López A, Caron JB. 2019 A possible case of inverted lifestyle in a new bivalved arthropod from the Burgess Shale. R. Soc. Open Sci. **6**, 191350. (10.1098/rsos.191350) 31827867 PMC6894550

[68] Fu D, Zhang X. 2011 A new arthropod *Jugatacaris agilis* n. gen. n. sp. from the early Cambrian Chengjiang Biota, South China. J. Paleontol. **85**, 567-586. 10.1666/09-173.1

[69] Fu D, Legg DA, Daley AC, Budd GE, Wu Y, Zhang X. 2022 The evolution of biramous appendages revealed by a carapace-bearing Cambrian arthropod. Phil. Trans. R. Soc. Lond. B **377**, 20210034. (10.1098/rstb.2021.0034) 35125000 PMC8819368

[70] Aria C. 2022 The origin and early evolution of arthropods. Biol. Rev. **97**, 1786-1809. (10.1111/brv.12864) 35475316

[71] Izquierdo-López A, Caron JB. 2021 A Burgess Shale mandibulate arthropod with a pygidium: a case of convergent evolution. Pap. Palaeontol. **7**, 1877-1894. (10.1002/spp2.1366)

[72] Boxshall GA. 2004 The evolution of arthropod limbs. Biol. Rev. Camb. Phil. Soc. **79**, 253-300. (10.1017/S1464793103006274) 15191225

[73] Edgecombe GD. 2017 Inferring arthropod phylogeny: fossils and their interaction with other data sources. Integr. Comp. Biol. **57**, 467-476. (10.1093/icb/icx061) 28957518

[74] Lozano-Fernandez J et al. 2019 Pancrustacean evolution illuminated by taxon-rich genomic-scale data sets with an expanded remipede sampling. Genome Biol. Evol. **11**, 2055-2070. (10.1093/gbe/evz097) 31270537 PMC6684935

[75] Walossek D. 1995 The Upper Cambrian *Rehbachiella*, its larval development, morphology and significance for the phylogeny of Branchiopoda and Crustacea. Hydrobiologia **298**, 1-13. (10.1007/978-94-011-0291-9_1)

[76] Pates S, Wolfe JM, Lerosey-Aubril R, Daley AC, Ortega-Hernández J. 2022 New opabiniid diversifies the weirdest wonders of the euarthropod stem group. Proc. R. Soc. B **289**, 20212093. (10.1098/rspb.2021.2093) PMC8826304 35135344

[77] Daley AC, Edgecombe GD. 2014 Morphology of *Anomalocaris canadensis* from the Burgess Shale. J. Paleontol. **88**, 68-91. (10.1666/13-067)

[78] Daley A, Budd GE, Caron JB, Edgecombe GD, Collins D. 2009 The Burgess Shale anomalocaridid *Hurdia* and its significance for early euarthropod evolution. Science **323**, 1597-1600. (10.1126/science.1169514) 19299617

[79] Kühl G, Briggs DEG, Rust J. 2009 A great-appendage arthropod with a radial mouth from the Lower Devonian Hunsrück Slate, Germany. Science **323**, 771-703. (10.1126/science.1166586) 19197061

[80] Edgecombe GD. 2020 Arthropod origins: integrating paleontological and molecular evidence. Annu. Rev. Ecol. Evol. Syst. **51**, 1-25. (10.1146/annurev-ecolsys-011720-124437)

[81] Briggs DEG. 1981 The arthropod *Odaraia alata* Walcott, middle Cambrian, Burgess Shale, British Columbia. Phil. Trans. R. Soc. Lond. B **291**, 541-582. (10.1098/rstb.1981.0007)

[82] Zhao Y et al. 2005 Kaili Biota: a taphonomic window on diversification of metazoans from the Basal Middle Cambrian. Acta Geol. Sin. English Ed. **79**, 751-756. (10.1111/j.1755-6724.2005.tb00928.x)

[83] Wen RQ, Zhao YL, Peng J. 2015 Morphology and ontogeny of *Tuzoia bispinosa* from the Kaili Biota (Cambrian Stage 5) of eastern Guizhou, China. Palaeoworld **24**, 61-70. (10.1016/j.palwor.2014.12.005)

[84] Caron JB, Jackson DA. 2006 Taphonomy of the greater phyllopod bed community, Burgess Shale. Palaios **21**, 451-465. (10.2110/palo.2003.P05-070R)

[85] Paterson JR, García-Bellido DC, Jago JB, Gehling JG, Lee MSY, Edgecombe GD. 2016 The Emu Bay shale Konservat-Lagerstätte: a view of Cambrian life from East Gondwana. J. Geol. Soc. Lond. **173**, 1-11. (10.1144/jgs2015-083)

[86] Sardà F, Company JB, Costa C. 2005 A morphological approach for relating decapod crustacean cephalothorax shape with distribution in the water column. Mar. Biol. **147**, 611-618. 10.1007/s00227-005-1576-y

[87] Lavalli KL, Spanier E. 2015 Predator adaptations of decapods. In The natural history of the crustacea, volume 2 lifestyles and feeding biology (eds M Thiel, L Watling), pp. 190-228. New York, NY: Oxford University Press.

[88] Scheltema RS. 1986 On dispersal and planktonic larvae of benthic invertebrates: an eclectic overview and summary of problems. Bull. Mar. Sci. **39**, 290-322.

[89] Wongkamhaeng K. 2004 Morphology and feeding ecology of gammarid amphipods in coral reef and seagrass communities. PhD thesis, Chulalongkorn University, Bangkok.

[90] Fryer G. 1977 Studies on the functional morphology and ecology of the Atyid prawns of Dominica. Phil. Trans. R. Soc. Lond. **277**, 57-129. (10.1098/rstb.1977.0007) 850693

[91] Barshaw DE, Spanier E. 1994 Anti-predator behaviors of the Mediterranean slipper lobster, *Scyllarides latus*. Bull Mar. Sci. **55**, 375-382.

[92] Haug JT, Caron JB, Haug C. 2013 Demecology in the Cambrian: synchronized molting in arthropods from the Burgess Shale. BMC Biol. **11**, 1-10. (10.1186/1741-7007-11-64) 23721223 PMC3685569

[93] Fatka O, Herynk J. 2016 The first report of the bivalved arthropod *Tuzoia* from the Skryje–Týřovice Basin (Barrandian area, Czech Republic). Ann. Paléontologie **102**, 219-224. (10.1016/j.annpal.2016.10.002)

[94] Spanier E, Lavalli KL. 2013 Commercial scyllarids. In Lobsters: biology, management, aquaculture and fisheries, 2nd edn (ed. BF Phillips), pp. 414-466. New York, NY: John Wiley & Sons Ltd.

[95] Nemoto T, Yoo KI. 1970 An amphipod, (*Parathemisto gaudichaudii*) as a food of the Antarctic Sei whale. Sci. Rep. Whales Res Inst. **22**, 153-158.

[96] Larsen K, Gutu M, Sieg J. 2015 Order Tanaidacea Dana, 1849. In Treatise on zoology-anatomy, taxonomy, biology The crustacea, volume 5 (eds JC von Vaupel Klein, M Charmantier-Daures, FR Schram), pp. 249-330. Leiden, The Netherlands: Koninklijke Brill NV.

[97] Kane JE. 1963 Observations on the moulting and feeding of a hyperiid amphipod. Crustaceana **6**, 129-132. (10.1163/156854063X00516)

[98] George RW, Main AR. 1967 The evolution of spiny lobsters (Palinuridae): a study of evolution in the marine environment. Evolution **21**, 803–820. (10.1111/j.1558-5646.1967.tb03435.x) 28563070

[99] Yang CH, Chan TY. 2012 On the taxonomy of the slipper lobster *Chelarctus cultrifer* (Ortmann, 1897) (Crustacea: Decapoda: Scyllaridae), with description of a new species. Raffles Bull. Zool. **60**, 449-460.

[100] Sheppard KA, Rival DE, Caron JB. 2018 On the hydrodynamics of *Anomalocaris* tail fins. Integr. Comp. Biol. **58**, 703-711. (10.1093/icb/icy014) 29697774

[101] Jin C et al*.* 2021 A new species of the Cambrian bivalved euarthropod *Pectocaris* with axially differentiated enditic armatures. Pap. Palaeontol. **7**, 1781-1792. (10.1002/spp2.1362)

[102] Izquierdo-López A, Caron JB. 2022 The problematic Cambrian arthropod *Tuzoia* and the origin of mandibulates revisited. Figshare. (10.6084/m9.figshare.c.6305917) PMC9727825 36483757

